# Dark Energy from Discrete Spacetime

**DOI:** 10.1371/journal.pone.0080826

**Published:** 2013-12-03

**Authors:** Aaron D. Trout

**Affiliations:** Department of Mathematics, Chatham University, Pittsburgh, Pennsylvania, United States of America; University of Oxford, United Kingdom

## Abstract

Dark energy accounts for most of the matter-energy content of our universe, yet current theories of its origin rely on radical physical assumptions such as the holographic principle or controversial anthropic arguments. We give a better motivated explanation for dark energy, claiming that it arises from a small negative scalar-curvature present even in empty spacetime. The vacuum has this curvature because spacetime is fundamentally discrete and there are more ways for a discrete geometry to have negative curvature than positive. We explicitly compute this effect using a variant of the well known dynamical-triangulations (DT) model for quantum gravity. Our model predicts a time-varying non-zero cosmological constant with a current value, 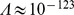 in natural units, in agreement with observation. This calculation is made possible by a novel characterization of the possible DT action values combined with numerical evidence concerning their degeneracies.

## Introduction

Multiple independent sets of empirical data [1]–[4] indicate that about 70% of the matter and energy in our universe comes from a mysterious repulsive gravitational effect known as “dark energy”. Understanding the origin of this energy is one of the most important problems in physics. Our only current theories involve speculative physical assumptions or finely tuned parameters. One popular assumption is the holographic principle: the idea that the degrees of freedom in a region of space are encoded on the region’s boundary [5]–[14]. Other explanations assume the existence of exotic matter fields or modify the Lagrangian defining general relativity. One recent theory [15] even hypothesizes a connection between dark matter and dark energy. See [16], [17] for reviews of various explanations of dark energy.

Our work provides a simpler, better motivated model for dark energy set within the well-known *dynamical triangulations* (DT) approach to quantum gravity. This model assumes no holographic principle, uses no additional matter fields or finely tuned parameters, and does not modify general relativity beyond the geometric discretization inherent in dynamical-triangulations spacetimes. In our model, a positive vacuum energy of the correct observed magnitude spontaneously arises from the entropic bias toward negative curvature states inherent in DT geometries. Note that treating gravity as an emergent mean-field phenomenon driven by entropic forces is a popular research perspective at the moment [18]–[24].

A reasonable prediction for dark-energy within a quantum-gravity theory is only significant if the theory approximates general relativity well at large distances. Why should we believe this about a theory that uses DT spacetime states? The progenitor of the DT theory, called the *Regge calculus*, has been used successfully in numerical general-relativity and quantum gravity for nearly five decades [25]–[31]. The DT model itself [32]–[36] and its descendent, *causal dynamical triangulations* (CDT) [32], [34], [35], [37]–[40] have been studied for nearly two decades. The numerous successes achieved by these theories give confidence that our model can describe general relativity at length-scales much larger than Planck’s length.

The model presented in this paper uses the same discretization of geometry and the same action as the DT theory. However, it is not identical to DT because it puts restrictions on the set of triangulations which contribute to the partition function. These kind of restrictions are also what distinguish DT from CDT although our restrictions are distinct from those in CDT. Note that it is not our purpose to advocate “triangulations” as the ultimate structure of spacetime. Indeed, in our calculation the discrete nature of geometry may be removed at the end without altering the predicted vacuum energy. We suspect that the effect described in this paper is actually a generic feature of any quantum-gravity theory which predicts a discrete spacetime geometry and which has general relativity as its large-distance limit.

## Background Material

General relativity can be written in the Lagrangian formalism using the Einstein-Hilbert action, which in natural units is
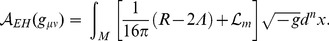
(1)


Here 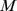 is a closed 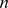-manifold, 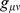 a Lorentzian metric, 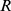 scalar-curvature, 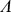 the cosmological constant, 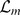 the Lagrangian for matter and 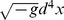 the standard volume element. See Table 1 for a list of commonly used symbols. Note, both 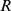 and 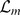 depend on 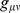 while 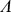 does not. Also note that 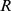 is the only term in this action with a physically distinguished zero value. In quantum field theory on a fixed background geometry, an arbitrary constant can be added to 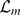 without changing the observed physics, allowing one to simply set 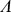 to zero. Thus, it is reasonable to argue, as we do in this paper, that the observed non-zero value of 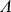 arises from quantum effects related to the scalar-curvature field 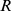.

**Table 1:** Meaning of Commonly Used Symbols.

Symbol	Meaning
*M*	closed *n*-manifold
*T*	triangulation of a closed *n*-manifold
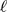	edge length of all edges in *T*
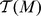	set of all triangulations of *M*
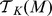	set of all triangulations of *M* with *K n*-simplices
*N_i_*(*T*)	number of *i*-simplices in a triangulation *T*
*μ*(*T*)	average hinge-degree of a triangulation *T*
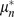	“flat” hinge-degree, 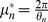 , 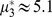 (irrational)
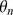	dihedral angle in a regular *n*-simplex, 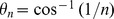
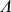	cosmological constant
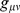	Lorentzian metric
*R*	scalar curvature of 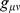
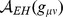	Einstein-Hilbert action
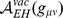	vacuum Einstein-Hilbert action with 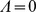
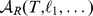	Regge action
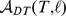	dynamical triangulations (DT) action
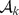	a DT-action in the 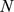 -action model
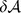	minimum separation between actions, see equation (14)
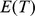	mean DT-action per volume for triangulation 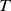
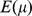	mean DT-action per volume at mean hinge-degree 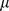
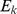	a mean DT-action in the 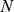 -action model
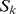	spacetime entropy in nats at mean-action 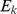
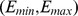	interval over which mean-actions are regularly spaced
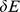	minimum separation between mean-actions, see equation (13)
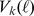	volume of 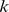 -simplex, all edge-lengths 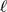 , 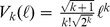
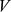	spacetime volume 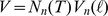
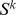	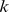 -dimensional sphere

In this table we list some of the commonly used symbols in this paper and their meanings.

Hilbert and Einstein showed that the critical points 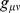 of this action satisfy the equations of motion
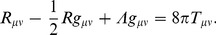
(2)


These are, of course, the field equations for general relativity. Here, 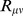 is the Ricci curvature tensor and 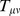 the stress-energy tensor for matter. In this work we restrict attention to the Einstein-Hilbert action for the vacuum with zero cosmological constant
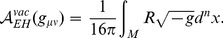
(3)


The critical points of 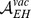 are metrics which satisfy the vacuum field equations. These metrics are *Ricci flat* everywhere (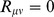 at every point) and therefore also *scalar flat* everywhere (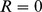 at every point.) Thus these metrics have action exactly zero. Finally, in dimensions less than four, the Ricci tensor determines the full curvature tensor 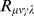, so critical points of 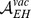 in these dimensions must actually be *flat* everywhere (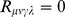 at each point.).

In his influential 1961 paper [25] Regge proposed a discretized version of 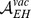 which applies to triangulated piecewise-linear (PL) manifolds. A **triangulation** 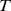 of a closed 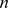-manifold 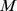 is a combinatorial 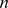-manifold homeomorphic to 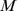 given as an abstract simplicial complex. Assigning a length 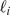 to each edge in 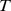 uniquely defines a piecewise-linear metric on 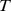 provided these lengths satisfy some natural compatibility conditions. If we let 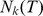 denote the number of 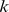-simplices in 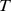, the **Regge action** is given by
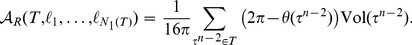
(4)


In this equation, the sum runs over all codimension-2 simplices of 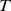 (called **hinges**), 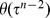 is the total dihedral angle around the hinge 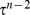, and 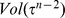 is that hinge’s volume. It is easy to insert a cosmological constant into this action, although here we do not. The possibility of incorporating matter fields into 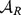 is a currently active topic of research. See [41]–[43].

Note that 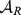 has a nice geometric interpretation. The summand in this action is the *angle defect* in a small triangle enclosing and perpendicular to the hinge 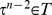, weighted by the volume of that hinge. Given the close relationship in classical non-euclidean geometry between angle defect and curvature, it is natural to interpret 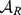 as a discrete measure of total curvature. Because of the success of the Regge action in describing general relativity, we will interpret 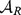 as a discrete measure corresponding to the Einstein-Hilbert action, and thus to **total scalar-curvature**. Interpreting 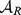 as a total curvature is also supported by the fact that, like point-wise curvature bounds in Riemannian geometry, bounds on the angle-defect for *all* hinges have profound topological consequences for 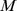. See [44]–[47] for examples.

### The Dynamical-Triangulation Action

Suppose we fix the abstract simplicial complex 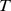 and consider 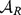 as a function of the edge-lengths 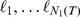 only. There is a large body of numerical evidence [26]–[29] that the critical points of this action define PL-metrics which behave like solutions 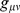 to the vacuum field equations, at least at length scales much larger than the maximum edge-length. See [36] for a overview of this work, known as the *Regge calculus*. In this paper, however, we will require all edges to have a single fixed length 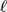 so that the action is determined only by the structure of 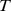 as an abstract simplicial complex, i.e. only on the way the simplices in 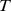 are attached together. This form of the Regge action has been studied extensively as part of the *dynamical triangulations* (DT) approach to quantum gravity. We write this action as
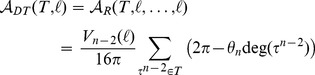
(5)where 
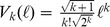
 is the volume of a 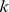-simplex with all edges of length 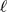, 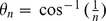 is the **dihedral angle** in such a simplex, and 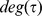, called the **degree** of 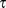, is the number of 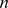-simplices in 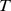 with 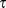 as a face. Usually, we will suppress the dependence on 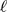 and write simply 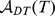.

Now for some terminology and preliminary results. Let 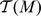 denote the set of all triangulations of a fixed closed 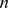-manifold 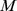. We will write 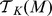 for the set of all triangulations of 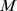 containing exactly 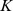 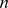-simplices, and 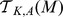 for those with 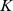 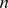-simplices and DT-action 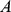. Since there are only finitely many ways to attach together the faces of a finite collection of 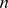-simplices, 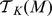 and 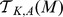 are finite sets. We define 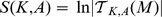 to be the **spacetime entropy** of 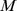 for 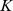 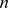-simplices and action 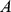. We will also need notation for the **average hinge degree** of a triangulation T,
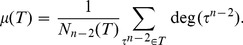
(6)


By double-counting arguments we may alternately write this as
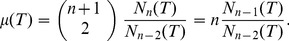
(7)


*Proof.* Suppose we examine each 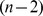-simplex 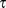 in 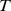 and place a mark on each 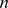-simplex with 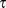 as a face. Clearly we have placed 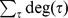 marks. On the other hand, each 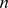-simplex has 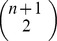 codimension-2 faces, so the number of marks is also 
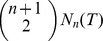
. Dividing through by 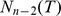 gives the first equality. Next, suppose we examine each 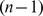-simplex 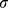 in 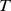 and place a mark on each of the two 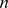-simplices incident at 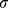. We have obviously placed 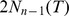 marks. However, each 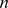-simplex has 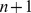 codimension-1 faces, so the number of marks is also 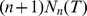 and we have 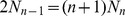. Plugging into the previous equality and simplifying finishes the proof.

The first part of equation (7) lets us nicely express 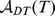 as a function of the number of 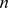-simplices in 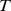 and its average hinge-degree. We get
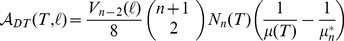
(8)where 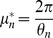 is called the **flat hinge-degree**. Why do we call 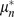 the *flat* hinge-degree? It is the number of regular 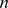-simplices needed around a hinge to provide a total dihedral angle of exactly 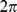, the expected quantity in a flat space. Note that, except in dimension two (where 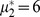) the quantity 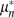 is not an integer.

*Proof of* *Equation (8* *).* We begin with the DT action (5) and distribute the sum into the summand to obtain
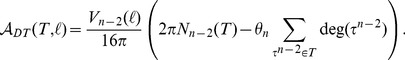
(9)


By equation (7) we can replace 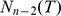 with 
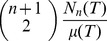
 and the summation by 
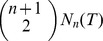
 to get
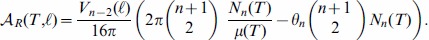
(10)


Finally, moving a factor of 
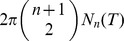
 to the front finishes the derivation.

### Mean Action Per Volume

The primary observable quantity of concern in this work is the **mean action per volume**, i.e. the average Lagrangian density over the manifold:
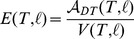
(11)where 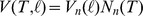 is the PL-volume of 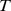. We use the symbol 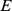 to remind us that this is a physically well-defined global observable with dimensions of energy per volume. Equation (8) gives a lovely formula for the mean action,
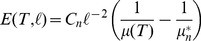
(12)where 
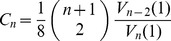
 depends only on the dimension 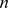. This tells us that for a fixed dimension and edge-length the mean-action depends only on the average hinge-degree 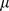. For notational convenience we will usually suppress the 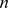 and 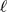 dependence and simply write 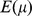 or 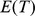.

Finally, note that for a fixed number of 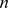-simplices 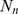 we can use equations (7) and (12) to find the minimum possible separation between mean-actions. This corresponds to changing the number of hinges by one, resulting in a change to 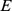 of
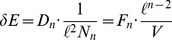
(13)where 
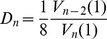
 and 
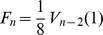
 depend only on the dimension 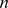 and 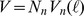 is the total spacetime volume. The minimum possible separation between actions is then given by
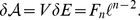
(14)


## Action Spectrum in Dimension Three

From this point forward, we will restrict attention to dimension three. What can we say about the possible values of 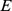 on 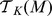 when 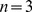
? This is a formidable problem, since even for a small number of tetrahedra 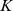 the set 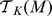 is quite large and complicated. We begin with an elementary result: for any triangulation 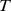 of a closed 3-manifold 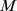 we have
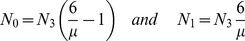
(15)where 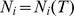 and 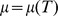. This means that for a fixed number of 3-simplices, the effect of increasing 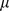 (or equivalently, decreasing 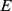) is to decrease both the number of vertices 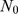 and the number of edges 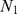 in the triangulation.

*Proof of* *Equation (15* *).* We begin with a well-known topological fact: every closed 3-manifold has Euler characteristic zero. That is, for any triangulation 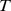 of a closed 3-manifold 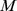 we have 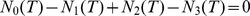. Now, we use equation (7) to replace 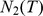 by 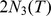 to get
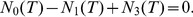
(16)


Using equation (7) again to replace 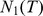 by 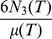 and then rearranging gives
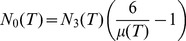
(17)as desired. Finally, we plug this 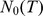 back into equation (16) and simplify to obtain
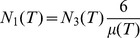
(18)completing the proof.

Equation (15) tells us that to understand the possible values for 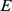 we must understand the possible combinations of 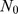 and 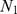 that can occur in a triangulation of a given closed 3-manifold. A 1970 paper [48] by Walkup tells us all we need to know.

**Theorem** (Walkup). *For every closed 3-manifold* 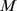 *there is a smallest integer* 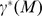 *so that any two positive integers* 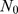 *and* 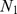 *which satisfy*

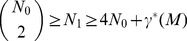
(19)are given by 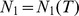 and 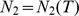 for some 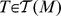. The quantity 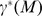 is a topological invariant which satisfies 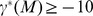 for all closed 3-manifolds 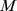.

Note that 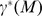 is known for many manifolds 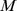, see [49], although we will not need this information.

Walkup’s Theorem, together with equation (15) and some algebra suffice to prove the central mathematical result in this paper:

**Theorem.** *Let* 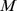 *be a closed 3-manifold and* 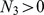 *a fixed number of tetrahedra. Then, there are mean actions*

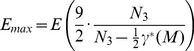
(20)and
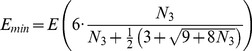
(21)so that if 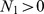 is an integer for which 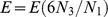 lies in the interval 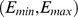 then 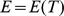 for some triangulation 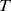 of 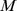 with 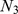 tetrahedra and 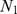 edges. These 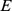 are regularly spaced over the entire interval 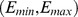, each separated from the next by
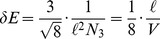
(22)where 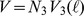. This is the smallest possible separation given fixed 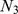, so these 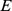 are all possible mean-actions on 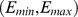.

Note that in most applications, the number of tetrahedra 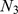 will be large and the energy densities given in the theorem will be approximately
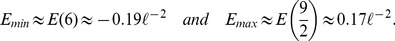
(23)


Also note that when edges are Planck’s length (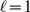 in our units) the magnitude of these energy densities is *enormous*, about 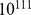 Joules per cubic meter.

*Proof of Main Theorem.* Let 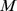 be a closed 3-manifold. We start by showing that if two given integers 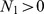 and 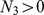 satisfy
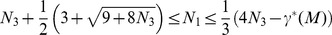
(24)then there is some triangulation 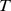 of 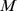 with 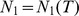 and 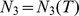. We define 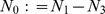. Note that 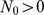 by the first inequality in (24). A bit of algebra applied to the second inequality in (24) implies



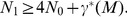
(25)Now, consider the upward opening parabola 
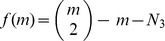
 which has largest root 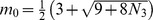. The first inequality in (24) implies 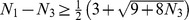 which is just 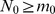. Since 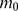 is the largest root of an upward opening parabola, we conclude 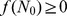. By our definition of 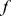 and 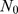, this tells us
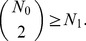
(26)


By Walkup’s theorem, inequalities (25) and (26) imply that some triangulation 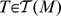 has 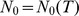 and 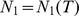. Finally, by equation (15), we know 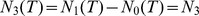 as desired.

Next, we divide the inequality (24) by 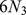 and take reciprocals to get
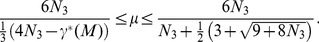
(27)where 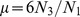. Thus, if 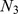 is fixed and 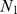 is an integer for which 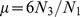 lies in this interval, then 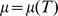 for some triangulation 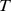 with 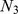 tetrahedra. By equation (12) the change in mean-action for each increment of 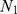 is as claimed in equation (22), completing the proof.

## The *N*-Action Model

The model used in this paper is designed to be dominated by states near a particular chosen target value 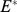 for the mean-action. For a fixed number of tetrahedra 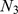 let 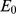 be the closest attainable mean-action to 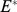. For each 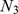, our model admits triangulations with mean-action 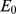 along with those having one of the 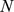 mean-action values on either side of 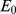. In this paper our target mean-action will be 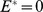 since the Einstein-Hilbert action for the vacuum in classical general-relativity is zero. Recall that, unlike actions in quantum field theory, the *values* of the Einstein-Hilbert and Regge actions are well-defined physical observables. This makes such a targeting strategy physically reasonable.

Why not simply start with a model containing only those triangulation 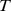 for which 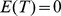
? It turns out that there are no such triangulations. That is, for any triangulation 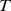 of a closed 3-manifold 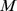 we have 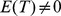, or equivalently 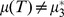. This follows from the irrationality of 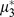 and equation (12). We know 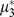 is irrational due to work [50] by Conway, Radin and Sadun on what are called called *geodetic angles*. Note that these angles are actually interesting mathematical objects on their own and are central to the solution to Hilbert’s third problem on the *scissor-congruence of polyhedra*.

So, let 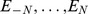 be the mean-actions in the model and 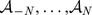 the corresponding total actions. Our main theorem implies that for any 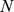 and spacetime volume 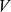 there is an 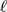 small enough so that all of the 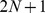 mean-action values 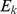 lie within the range 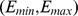 where attainable action-values are regularly spaced. For such 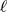 our model has partition function
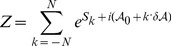
(28)where 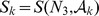 is spacetime entropy at action 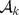. The expected action for this model is then



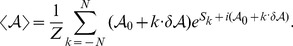
(29)A Euclidean version 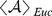 of this expected value can be found by applying the standard Wick rotation 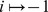 to the expression above.

It is currently impossible to write 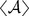 or 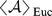 as exact closed form expressions since the entropies 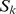 are beyond our ability to compute. However, if we replace 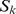 with its first order approximation 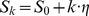 for 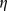 a constant, then a closed-form expression can be found. We used the computer-algebra package Mathematica to show
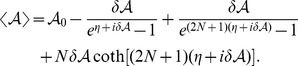
(30)


A closed form expression for 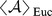 can obtained as before by replacing 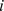 with 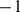 in the equation above.

### Choosing 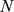


How are we to choose 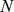
? In an ideal world, we would have in hand a fully formed DT-style theory of quantum gravity coupled to matter, which provably reduced to general-relativity at large distances. From this theory we could *derive* an appropriate 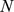 by computing how far a typical spacetime was from the classical action. We believe such a theory will eventually emerge, but it is not yet available. However, we have set up enough machinery to reasonably guess what such a theory would tell us about 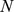.

Suppose we fix a total spacetime volume 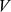 and consider the 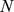-action theory targeting mean-action zero. What happens as we let the edge-length 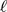 approach zero? Because the separation between actions 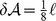 goes to zero and 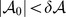, if 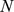 is left fixed as 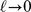 then even the most extreme action values in the theory, 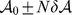, would converge to zero. Since we wish to investigate *quantum* gravity, this is unacceptable and we are forced to choose an 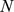 which diverges as 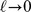. Now, suppose we make the affine entropy approximation 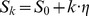. Equation (30) implies that if 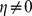 then for large enough 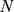 and small enough 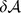 the expected action is dominated by the final hyperbolic cotangent term and we have 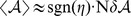. This tells us that under these conditions, the model is completely dominated by entropy. The oscillating complex phase 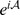 which suppresses the contribution of states far from 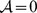 is swamped by the entropy term involving 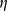.

Thus, since 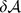 is proportional to 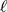, it is natural to choose the dimensionless 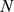 to be proportional to 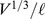. For such a choice we can take the 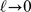 limit and the theory gives a finite non-zero value for the expected action. Therefore, we choose to use
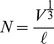
(31)mean-action values on either side of 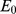. Notice that by the approximations (23) even though 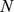 diverges as 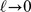, all actions in the model eventually lie within the “regularly spaced” range 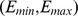 for small enough 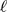. Also note that as 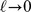 all the mean edge-degrees corresponding to these 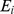 converge to the flat mean edge-degree 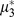.

Finally, for any fixed 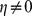 we can use equation (30) to compute the 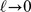 limit, obtaining
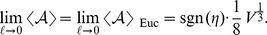
(32)


For 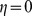 we get a purely imaginary standard expectation 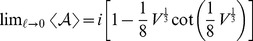 and a Euclidean expectation given by 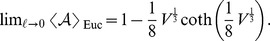


## Evidence for the Entropies 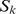


The calculation of the expected action as 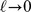 given by equation (32) depends on two assumptions about the entropies 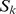. First, for the states contributing to the model, spacetime entropy must be an approximately linear function of mean-action, i.e. 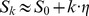, at least for large enough 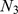. Second, this 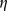 must not approach zero as 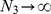. In this section, we present evidence from Monte-Carlo simulations and small-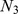 enumerations that strongly supports these assumptions.

### Monte-Carlo Sampling Results

To measure the dependence of entropy on mean-action we use a Metropolis-Hastings algorithm to take samples 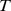 from 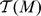 near a given number of tetrahedra and mean-action. The algorithm wanders among the elements of 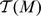 by using the well-known *Pachner moves* to change from one triangulation to another, repeatedly choosing a random move and executing it with probability 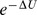 where 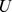 is some non-negative objective function. Metropolis proved that if we wait long enough between samples, then each sample 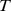 occurs with probability 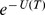. Here, we use a quadratic objective function
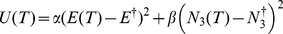
(33)with 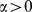 and 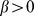 fixed constants. This form for 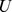 keeps the sampled triangulations near a target mean-action 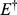 and number of tetrahedra 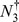.

If there were equally many triangulations at each 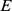 and 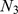 then our sampled pairs 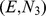 would form a Gaussian distribution centered at the target point 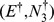. If our samples have a Gaussian distribution but with mean 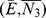 significantly displaced from the target, this indicates a *linear* dependence of spacetime entropy on 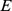 and 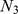 with the magnitude of the dependence proportional to the size of this displacement. Since it is obvious that spacetime entropy is strongly dependent on 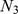 and because the relative deviation from the mean for 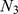 is at most 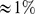 in our data, we focus solely on deviation in mean-action 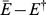. From this we can estimate the change entropy (per mean-action step) 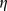, in nats, using
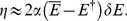
(34)


**Figure 1:**
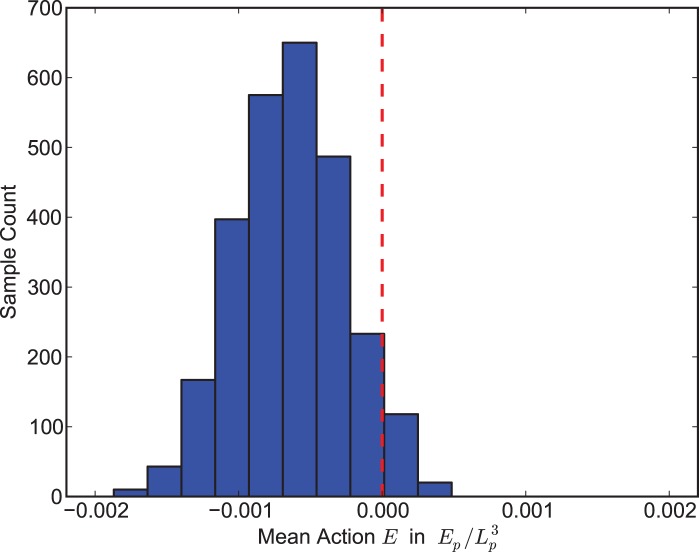
Monte-Carlo sampling of triangulations of 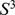 near mean-action zero. We plot the distribution of mean actions 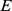 at 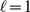 for 2700 sampled triangulations of the 3-sphere 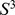. Samples were obtained from a Metropolis-Hastings algorithm using Pachner moves and a quadratic objective function 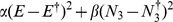 targeting 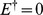 and 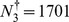 with 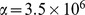 and 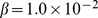. Waiting times were chosen so that 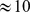 accepted moves per tetrahedra occurred between successive samples. Observed means were 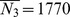 with standard deviation 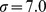 and 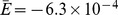 with standard deviation 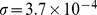. Note that 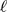 and 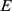 are given in Planck units, 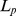 and 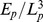 respectively.

**Figure 2:**
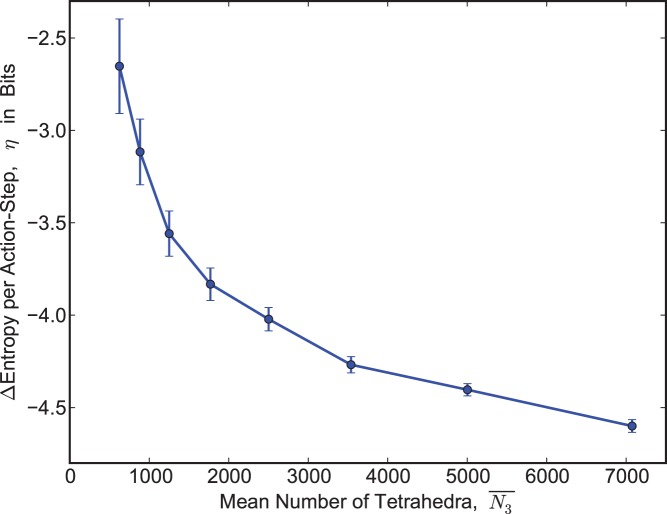
Entropy remains a decreasing function of mean-action as the number of tetrahedra grows. We plot the change in spacetime entropy, 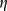 in bits, due to each minimal increase 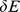 in mean-action for the 3-sphere 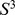 near 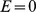, versus mean number of tetrahedra 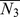. Values were inferred from the bias seen in Monte-Carlo samples of triangulations near 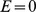. See Figure 1. All data points except the last two were computed from 2700 samples. At the two largest 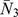 values, we used 2394 and 1108 samples respectively. Error bars indicate 95% confidence intervals.

The sampling trials conducted for this paper use the 3-sphere 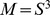 with target mean-action zero (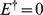) and various targets for the number of tetrahedra 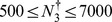. In all cases, we take 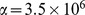 and 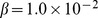. In order to ensure independent samples, the algorithm attempts Pachner moves until 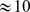 accepted moves per tetrahedron have occurred. We checked that this wait time was sufficient using standard correlation tests. For these parameters, each sample was uncorrelated from the next. We also checked that the sampled 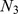 and 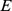 were independent. As desired, samples are approximately normally distributed with sample mean 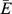 somewhat displaced from the target 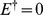. This indicates that entropy is approximately a linear function of mean-action near 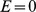, as was assumed in the previous section. See Figure 1 for a histogram of mean-actions for 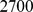 samples at 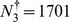. For each such distribution, we use equation (34) to infer the approximate change in entropy 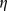 between mean-actions. These 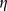 are comfortably negative and do not appear to approach zero as 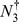 gets larger, validating our second assumption. See Figure 2. Copies of the code used for triangulation sampling are available on request.

### Triangulation Census Data

**Figure 3:**
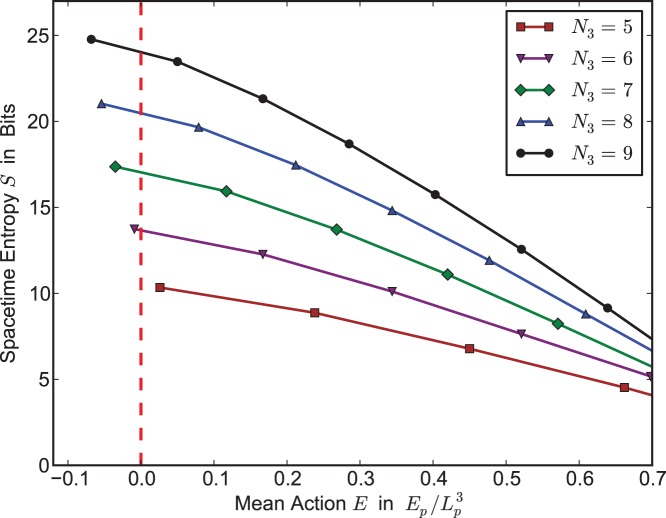
Entropy versus mean-action from triangulation census data. We plot spacetime entropy 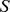 in bits for the three-sphere 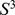 at various numbers of tetrahedra 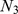, versus mean action 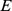 at 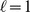. Data come from a complete census [51], [52] of the 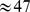 million triangulations of 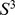 with at most 9 tetrahedra. Note that 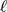 and 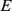 are given in Planck units, 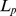 and 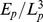 respectively.

In addition to Monte-Carlo sampling evidence, one can also see a bias towards negative action states in computer-generated censuses of 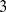-manifolds triangulations. In particular, recent advances in enumeration algorithms have allowed for the creation of an explicit list of all triangulations of any closed 3-manifold using at most 11 tetrahedra. See [51], [52]. Unfortunately, the definition of a “triangulation” used in these censuses is slightly more general than ours. They define a triangulation of a closed 3-manifold 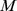 as a space homeomorphic to 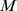 obtained by identifying the faces of some finite set of tetrahedra. We believe this to be a largely technical distinction, and we expect this data to provide a good guide to the general features of our set of triangulations 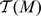. See Figure 3 for a graph of spacetime entropy versus mean-action for the 3-sphere 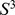 and 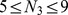. We observe two trends in the data. First, as we expect, the number of triangulations increases as the number of 3-simplices grows. However we also see the same effect as observed in the Monte-Carlo sampling experiments: the number of triangulations at a given action is a *decreasing function of action*.

## The Origin of Dark Energy

Taking 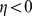 and dividing through by 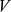 in equation (32) gives
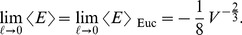
(35)


Let us briefly discuss the physical meaning of 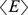. Our goal was to construct a theory dominated by states close to the classical value of the mean-action, 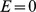. We did this by “slicing” the partition function according to action-value, retaining only states whose actions lie within a certain distance of zero. If the volume of spacetime is large compared to Planck’s volume then we come very close to accomplishing our goal. That is, for 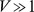 we do indeed obtain 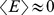 in the 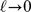 limit. However, there *is* a small perturbation away from zero because of the relative entropy of action values. Notice that since action values are global observables, this effect is independent of the local details of the “metric”, i.e. the local structure of the triangulation. This leads us to expect that, for a typical triangulation at a given 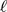, the average action will appear very uniform at length-scales much larger than 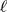. Finally, recall that everything in the Einstein-Hilbert action *except* the cosmological constant 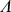 depends on the metric 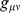. Thus, the basic structure of 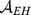 almost demands we interpret our non-zero 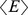 as an emergent cosmological constant given by
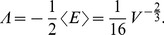
(36)


We now turn to the question of applying this result to our own universe. This is a somewhat speculative endeavor since our world appears to be both 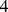-dimensional and infinite in extent. However, as an entropic effect connected with the pattern of attachment between simplices, we expect the perturbation away from 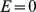 identified in this paper to occur quite generally. So, what 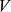 is appropriate for assessing the magnitude of this effect in our particular universe? Considerations of causality give us a reasonable answer: take the volume of space which has had time to causally communicate with our point of observation. That is, we ought to use something like the current *Hubble volume* 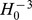 where 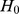 is the Hubble constant. Plugging in 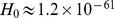 in Planck units gives
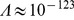
(37)


which is in general agreement with observation.

At this point, we feel obliged to briefly discuss the term “numerology”. It has long been known that the observed cosmological constant was approximately 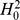. This and many other unexplained approximate numerical relationships between cosmological parameters are often called *large number coincidences*. Thinking of them as having explanatory power on their own is surely deserving of the label “numerology”. However, this epithet should not be applied to a physically well-motivated theory which predicts *ab-initio* such a numerical relationship, as our model does.

## Discussion

Our derivation of 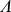 has some interesting features. Using the Hubble parameter to define our characteristic volume 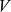 means that the model actually predicts a time-varying cosmological constant
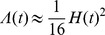
(38)


where 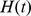 is the Hubble parameter at proper time 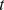. That is, we predict that 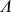 scales like the area of the cosmic horizon. Amazingly, although we made no holographic assumption, this is the same behavior that emerges from *holographic dark energy* (HDE) theories [5], [6], [10], [12], [13]. In fact, our model shares several other key features with these approaches, including the presence of two “cut-offs” in the theory which are removed in a coordinated fashion. HDE models typically contain both a UV and IR field cut-off which are removed in a way that saturates entropy in the Bekenstein bound. In our theory, the cut-offs 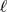 and 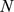 are chosen to keep the entropic perturbation on 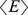 bounded as 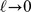. While HDE theories are very different in detail from our model, the broad similarities are quite striking. Perhaps both approaches are pointing to the same underlying physical issues. We hope that the relative simplicity of our model can help elucidate these issues.

We should also mention another explanation for 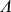 which shares some features with our approach. In [53] it is argued that the true ground-state vacuum has 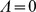 but that we observe 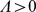 because the universe has not yet had time to decay into this ground state. The author considers a model in which the true ground state is given by the superposition of two degenerate 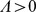 states, one of which describes the universe’s present-day vacuum. Since the decay probability in a given volume and time period is related to the energy density 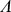, the requirement that no such decay has yet happened in the Hubble volume provides an estimate for 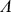 which agrees with observation. This argument leads, as does our model, to a connection between the Hubble parameter and 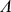. Also note that both models contain states at or near 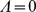 which are suppressed compared to the 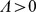 states.

Finally, we note that in the very early universe our model predicts large 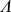 and hence rapid expansion. This raises the tantalizing possibility that big-bang inflation and dark-energy are manifestations of a common effect, though it is likely that a more sophisticated choice for the characteristic volume 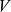 would be needed. See [54] for consideration of this idea in the HDE context.
