# Correction: Dark Energy from Discrete Spacetime

**DOI:** 10.1371/annotation/ddb13efe-97bf-4a70-ad3b-a11fb2eb23f2

**Published:** 2014-01-29

**Authors:** Aaron D. Trout

This article was republished on January 23, 2014 because of illegible mathematical symbols. Please download this article again to view the symbols. The originally published, uncorrected article is provided here for reference: http://plosone.org/corrections/pone.0080826.cn.pdf

